# Draft Genome Sequence of *Halobacillus* sp. Strain KGW1, a Moderately Halophilic and Alkaline Protease-Producing Bacterium Isolated from the Rhizospheric Region of *Phragmites karka* from Chilika Lake, Odisha, India

**DOI:** 10.1128/genomeA.00361-16

**Published:** 2016-06-30

**Authors:** Ananta Narayan Panda, Samir R. Mishra, Lopamudra Ray, Neha Sahu, Ankita Acharya, Sudhir Jadhao, Mrutyunjay Suar, Tapan Kumar Adhya, Gurdeep Rastogi, Ajit Kumar Pattnaik, Vishakha Raina

**Affiliations:** a School of Biotechnology, KIIT University, Bhubaneswar, Odisha, India; b Wetland Research and Training Center, Chilika Development Authority, Department of Forest and Environment, Bhubaneswar, Odisha, India; c Bionivid Technology Private Limited, Kasturi Nagar, Bangalore, India

## Abstract

*Halobacillus* sp. strain KGW1 is a moderately halophilic, rod shaped, Gram-positive, yellow pigmented, alkaline protease-producing bacterium isolated from a water sample from Chilika Lake, Odisha, India. Sequencing of bacterial DNA assembled a 3.68-Mb draft genome. The genome annotation analysis showed various gene clusters for tolerance to stress, such as elevated pH, salt concentration, and toxic metals.

## GENOME ANNOUNCEMENT

*Halobacillus* species are Gram-positive spore-forming bacteria having low G+C content, as identified by Spring et al. (1). Including the other type species *Halobacillus halophilus* (formerly *Sprorosarcina halophile*), *Halobacillus littoralis* (DSM 10405^T^) and *Halobacillus truperi* (DSM 10404 ^T^) (2), the *Halobacillus* genus is composed of 21 species that are validly published. This genus features pigment-producing (3) moderate halophiles that are capable of producing a variety of enzymes at a wide range of salinity and pH (4). Thus, halophiles are suitable species for screening of enzyme and carotenoid production useful for various industrial applications. Chilika Lake is situated between 19°28′ and 19°54′N latitude and 85°05′ and 85°38′E longitude. *Phragmites karka* (a common reed) forms monoculture dense patches in lagoon Chilika and is considered to be a highly invasive weed in Chilika Lake (5, 6).

The microbial communities associated with the rhizospheric region of these macrophytes contribute toward overall biogeochemical cycling and play an important role in nutrient cycling at the ecosystem level (7). *Halobacillus* sp. strain KGW1^T^ was isolated from rhizospheric region water sample of macrophyte-dominated areas at Kalupara Ghat, Chilika Lake (19.84699′N and 85.40778′E) by dilution plating technique at 30°C on Zobell’s marine broth (ZMB; HiMedia, India). It is a Gram-positive, alkaline protease-producing bacterium that can grow from a temperature 15°C to 45°C and can tolerate alkaline pH (pH 5.5 to 11.5) and salt (0 to 25% [wt/vol] NaCl).

Genomic DNA was extracted using the Gnome kit (MP Biomedicals, Santa Ana, CA). The genome sequence of strain KGW1^T^ was sequenced using an Illumina MiSeq sequencing platform. The data generated were assembled using the Velvet (version 1.2.10) assembler (8), resulting in 13 contigs, out of a total of 3,683,719 bp, and an *N*_50_ contig size of 1,191,089 bp (1.19 Mb). The estimated complete genome size was 3.68 Mb, with a G+C content of 46.98%. Genome annotation was performed using the Rapid Annotations using Subsystems Technology (RAST) (9, 10), which predicted a total of 3,900 protein-coding sequences, 74 pseudogenes, 67 tRNAs, and 7 rRNA clusters. The taxonomy identification was performed using EzTaxon and MEGA6, which identified *H. trueperi* as the putative species per 16S rRNA gene sequence homology. PHAST analysis (11) revealed a putative intact phage integrated in the genome, with a length of 54.3 kb, 52 protein-coding sequences, and a G+C content of 42.36%.

The RAST annotations identified various major gene clusters for stress regulation, resistance to toxic compounds and heavy metals, protein degradation, carbohydrate degradation, degradation of multiple aromatic compounds, lipid metabolism, 1-aminocyclopropane-1-carboxylate deaminase activity, auxin biosynthesis, nitrogen, metabolism, siderophore production, phosphorous solubilization, and sulfur metabolism.

### Nucleotide sequence accession numbers.

This whole-genome shotgun project has been deposited in DDBJ/EMBL/GenBank under the accession no. LSOC00000000. The version described in this paper is the first version, LSOC01000000.
